# Dietary Supplement and Prescription Medication Use among Older Adults with Cognitive Impairment.

**DOI:** 10.1093/geroni/igaf122.2576

**Published:** 2025-12-31

**Authors:** Hao-Ping Chiang, Brady West, Antoinette Coe

**Affiliations:** University of Michigan, Ann Arbor, Michigan, United States; University of Michigan, Ann Arbor, Michigan, United States; University of Michigan, Ann Arbor, Michigan, United States

## Abstract

Supplement use among older adults is associated with higher prescription medication use, leading to medication harm and hospitalizations. As patients with cognitive impairment are vulnerable to polypharmacy and these adverse outcomes, it’s essential to understand how supplement use contributes to medication burden. This cross-sectional study examined the association between dietary supplement and prescription medication use among U.S. adults aged 60 and older with cognitive impairment, using the National Health and Nutrition Examination Survey (NHANES). NHANES 2011–2014 was selected as the only publicly available period with objective cognitive measurements (Cognitive Functioning Assessment) for defining impairment. The outcome was the number of prescription medications. The primary predictor was dietary supplement use (yes/no). Covariates included age, sex, race, education, insurance, and comorbidities. Mean and proportion differences between supplement users and non-users were analyzed using design-adjusted t-tests and Rao–Scott tests. Negative binomial regression models evaluated associations between the predictor variables and the number of prescription medications. Among an estimated 20,939,624 older adults with cognitive impairment (based on 1,567 NHANES respondents), an estimated 65.6% used dietary supplements. Supplement users took more prescription medications on average than non-users (4.8 vs. 4.2, p = 0.036). Supplement use was significantly associated with increased prescription medication use (estimated rate ratio = 1.14, 95% CI 1.01–1.29), but not after adjustment for covariates. Given the adverse outcomes of increased medication use, recognizing supplement use in older adults with cognitive impairment is suggested for medication safety. Further research is needed to clarify supplements’ role in medication burden.

